# Anti-sliding plate technique for coronal shear fractures of the distal humerus

**DOI:** 10.1186/s13018-019-1466-5

**Published:** 2020-01-17

**Authors:** Zhe Song, Qian Wang, Teng Ma, Chen Wang, Na Yang, Hanzhong Xue, Zhong Li, Yangjun Zhu, Kun Zhang

**Affiliations:** Department of Orthopaedic Trauma, Hong-Hui Hospital, Xi’an Jiaotong University, No. 76, Nanguo Road, Beilin District, Xi’an City, 710054 Shaanxi Province China

**Keywords:** Fracture, Distal humerus, Elbow joint, Internal fixation, Technique

## Abstract

**Purpose:**

The purpose of this study is to discuss the surgical strategy, technical feasibility, and clinical efficacy of coronal shear fractures of the distal humerus using the anti-sliding plate technique.

**Methods:**

Fifty-two patients (35 males and 17 females) were treated with the anti-sliding plate technique in our hospital from January 2012 to January 2017. The average age of the patients was 40.4 years. They were classified according to the Dubberley classification system and treated with the anti-sliding plate technique. The long-term functional scores represented by the Mayo Elbow Performance Index and complications were evaluated.

**Results:**

Fractures were classified as follows: 11 type-IA, 5 type-IB, 16 type-IIA, 4 type-IIB, 13 type-IIIA, and 3 type-IIIB according to the Dubberley classification system. All patients were treated with open reduction and internal fixation by the extensile lateral approach and completed a clinical and radiographic follow-up (average, 17.6 months). The average Mayo elbow performance score was 90.6 points, with 36 excellent, 11 good, and 5 fair results. The average range of movement of the elbow joint was 3° (0–15°) for extension and 136° (90–150°) for flexion.

**Conclusions:**

The anti-sliding plate technique follows basic AO principles and neutralizes the shearing force combined with lag screws and/or Kirschner wires after the anatomic reduction of the fracture. It allows for the stable internal fixation of the fracture, which is critical for early mobilization and a good functional outcome.

**Level of evidence:**

Level IV, Case Series, Treatment Study

## Introduction

Coronal shear fractures of the distal humerus affect the articular surfaces of the capitellum and/or trochlea. This type of fracture is rare, causing intra-articular damage of the distal humerus and accounting for less than 1% of elbow fractures [1, 2]. This type of fracture is also likely to be misdiagnosed. However, the complexity of these fractures in recent times has been better appreciated by digital imaging and computed tomographic scans [3]. Treatment strategies for these injuries have evolved over time from conservative management to open surgery. Currently, open reduction and internal fixation are the preferred methods for treating this type of injury [4].

Given the rarity of coronal shear fractures, it has been difficult to formulate a universally accepted method of fixation [5]. Various treatment methods have been proposed, but no final conclusion has been drawn [6]. Several authors have recommended fragment excision, but this approach can result in pain, instability, and loss of motion [7, 8]. Kirschner wires and bio-absorbable screws have also been used with less favorable results, as they fail to provide sufficient fixation strength [9]. Therefore, most reports in the literature recommend fixation with cancellous lag screws or Herbert’s screws placed in the anterior to posterior direction [7, 10, 11]. However, the placement of the screws can damage a considerable amount of articular cartilage, which cannot resist the shear force on the coronal plane. Furthermore, the fracture fragments provide limited access for screw placement, while trying to achieve stable fixation. In this article, therefore, we discuss a technique for the internal fixation of coronal shear fractures of the distal humerus using the anti-sliding plate combined with lag screws and/or K-wires. We have used this technique to treat 52 patients from January 2012 to January 2017. Here, we aim to report on the demographic characteristics, as well as the clinical and radiographic outcomes, with a minimum follow-up time of 1 year.

## Materials and methods

### Materials

This was a retrospective study of coronal shear fractures of the distal humerus. Fifty-two patients were diagnosed and managed operatively for coronal shear fractures of the distal humerus at our institution from January 2012 to January 2017. They were subsequently followed clinically and radiographically with subjective and objective outcome measures. No patient was excluded. The study was approved by the Ethics Review Committee of Hong-Hui Hospital, Xi’an Jiaotong University College of Medicine, Shaanxi Province, China. Informed consent was obtained from all participants included in the study (Table 1).

**Table 1:** Main characteristics and Follow-up results of the study population

Patient	Gender	Age	Type of trauma	Type of fracture	Type of fracture	Mayo elbow performance score	Follow-up duration
ID		(year)		(Dubberley classification)	(AO/OTA classification)	(MEPS)	(month)
1	M	29	Fall from the ground	IA	B3.1	Excellent	18
2	F	37	Fall from the roof	IIIA	B3.3	Fair	15
3	M	34	Fall from the bike	IIA	B3.3	Excellent	18
4	M	31	Fall from the stairs	IIB	B3.3	Good	24
5	M	35	Fall from the ground	IA	B3.1	Excellent	12
6	F	53	Fall from the stairs	IIA	B3.3	Excellent	20
7	M	36	Fall from the bike	IIIA	B3.3	Good	16
8	F	47	Fall from the ground	IB	B3.3	Excellent	20
9	M	62	Fall from the ground	IA	B3.1	Excellent	24
10	M	31	Fall from the bike	IIA	B3.3	Excellent	12
11	F	27	Fall from the stairs	IIIA	B3.3	Good	12
12	F	41	Fall from the ground	IA	B3.1	Excellent	24
13	M	32	Fall from the ground	IIIA	B3.3	Excellent	16
14	M	27	Fall from the stairs	IIA	B3.3	Excellent	12
15	F	55	Fall from the ground	IIA	B3.3	Excellent	18
16	M	38	Fall from the bike	IIB	B3.3	Good	15
17	M	44	Fall from the ground	IA	B3.1	Excellent	12
18	F	36	Fall from the ground	IIA	B3.3	Excellent	15
19	M	27	Fall from the ground	IIA	B3.3	Excellent	24
20	M	56	Fall from the ground	IA	B3.1	Excellent	18
21	M	27	Fall from the bike	IIIA	B3.3	Good	18
22	F	37	Fall from the ground	IB	B3.1	Excellent	16
23	M	54	Fall from the ground	IIA	B3.3	Excellent	20
24	M	34	Fall from the roof	IIIA	B3.3	Good	21
25	M	38	Fall from the stairs	IA	B3.1	Excellent	14
26	F	61	Fall from the ground	IIA	B3.3	Excellent	15
27	M	48	Fall from the bike	IIIA	B3.3	Good	18
28	F	32	Fall from the ground	IIA	B3.3	Excellent	16
29	M	43	Fall from the ground	IA	B3.1	Excellent	18
30	M	33	Fall from the ground	IIIA	B3.3	Excellent	14
31	F	34	Fall from the roof	IIIB	B3.3	Fair	12
32	F	41	Fall from the ground	IIA	B3.3	Excellent	20
33	M	43	Fall from the stairs	IIIA	B3.3	Good	15
34	M	32	Fall from the stairs	IIA	B3.3	Excellent	16
35	F	35	Fall from the ground	IA	B3.1	Excellent	20
36	M	48	Fall from the ground	IIIA	B3.3	Good	24
37	M	49	Fall from the ground	IIA	B3.3	Excellent	16
38	F	39	Fall from the stairs	IIIB	B3.3	Fair	24
39	M	45	Fall from the ground	IIIA	B3.3	Excellent	14
40	M	56	Fall from the ground	IIA	B3.3	Excellent	18
41	M	61	Fall from the ground	IB	B3.1	Excellent	20
42	M	42	Fall from the bike	IIIA	B3.3	Excellent	18
43	F	51	Fall from the ground	IIA	B3.3	Excellent	20
44	M	37	Fall from the roof	IIIB	B3.3	Fair	24
45	M	53	Fall from the ground	IA	B3.1	Excellent	16
46	F	27	Fall from the ground	IIB	B3.3	Good	18
47	M	34	Fall from the ground	IB	B3.1	Excellent	14
48	M	29	Fall from the bike	IIIB	B3.3	Fair	18
49	M	30	Fall from the stairs	IIIA	B3.3	Good	20
50	F	46	Fall from the ground	IA	B3.1	Excellent	12
51	M	35	Fall from the ground	IIA	B3.3	Excellent	22
52	M	49	Fall from the ground	IB	B3.1	Excellent	20

Details on the injury type, specifically the results of the clinical examination and radiographic tests, were recorded for each patient. The coronal shear fractures of the distal humerus were classified according to the AO/OTA classification system [12] and Dubberley classification system [13]. Radiographs were obtained immediately after the injury (anteroposterior and lateral views), and CT scans were reviewed by three experienced orthopedic surgeons. Fractures were classified based on routine radiographs, and the fracture type was confirmed with intraoperative findings.

All patients were followed up every month until the sixth postoperative month, and then annually until the most recent follow-up. The follow-up evaluation included clinical and radiographic assessments. The clinical functional assessment included the Mayo elbow performance score (MEPS) [14], the range of flexion and extension of the elbow joint, the rotation of the forearm, and the stability of the elbow joint. The radiographic assessment included an evaluation of the fracture reduction (anatomic, near anatomic [less than 2 mm displaced], or displaced [greater than 2 mm]) [15], fracture union, osteonecrosis, heterotopic ossification (classified by the Brooker system and applied to the elbow) [16], and post-traumatic osteoarthritis according to the Broberg and Morrey classification system [17].

### Surgical technique

**Fig. 1:**
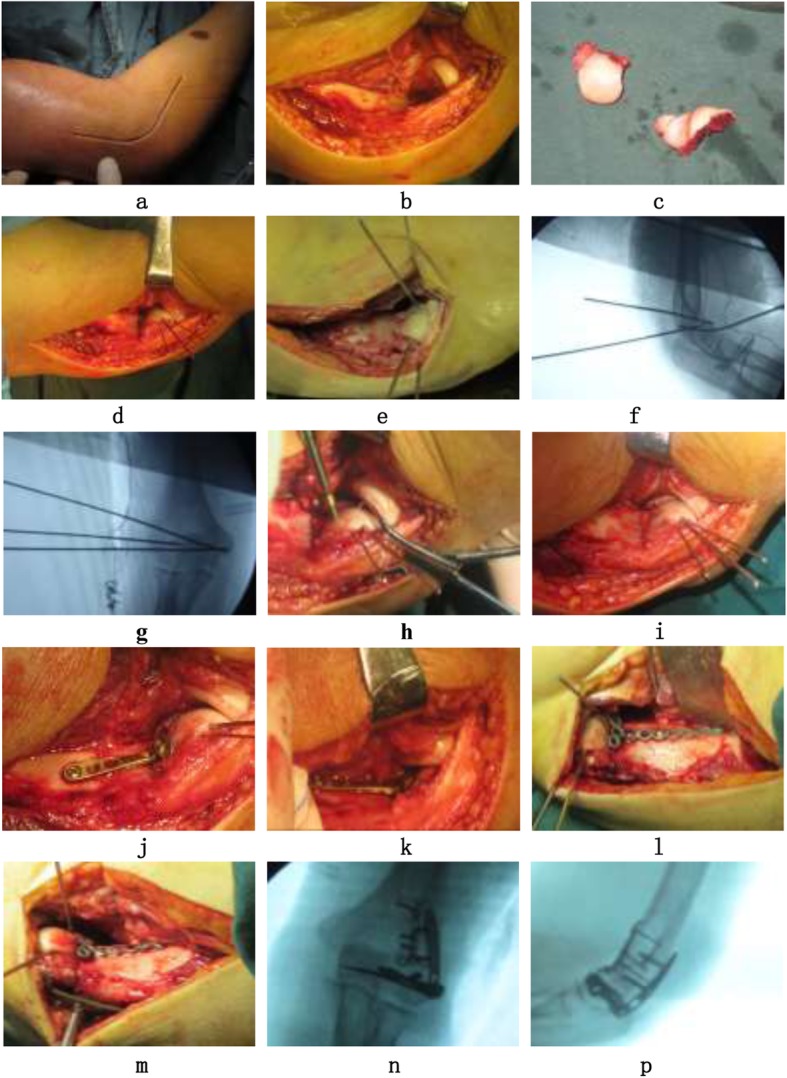
Surgical procedures: extensile lateral approach (**a**), dissect and expose the fracture site (**b**), the fragment is comminuted and dissociative sometimes (**c**), “on-table” technique was used for this type of fractures (**d**), temporary fixation with Kirschner wires after the fracture anatomical reduction (**e**, **f**), observe the quantity of fracture reduction with intraoperative fluoroscopy (**g**, **h**), countersunk screws were used to fixate from the anterior side to the posterior side of articular surface (**i**, **j**), mini-plate were adhered beneath the articular surface of the capitellum for the anti-sliding fixation, without the of the obstruction of humeroradial joint (**k**, **l**), use two mini-plate for some Dubberley B type fractures, one is placed on anterolateral side and another is placed on posterolateral side (**m**, **n**), intraoperative fluoroscopy is used to determine the fracture reduction again and the placement of implants (**o**, **p**)

All cases were operated on under general anesthesia in the supine position with a bump underneath the scapula, and the arm brought across the chest. A tourniquet was used in all cases. The extensile lateral approach to the elbow was used, centered over the lateral epicondyle. The interval between the anconeus and the extensor carpi ulnaris was identified distally, and the common extensor origin and the extensor carpi radialis brevis were elevated anteriorly, which made it possible to reach and elevate the anterior capsule of the elbow joint. Thus, the capitellum and trochlea of the humerus were exposed directly. The front part of the entire articular surface was exposed with a Hoffman retractor on the medial side of the distal humerus at a 30–45° flexion position of the elbow joint. Firstly, all the fracture fragments were reduced to their normal anatomical positions with K-wires and/or headless screws from the anterior to the posterior position. Secondly, the remodeled T type microplate was placed beneath the articular surface of the capitellum as the anti-sliding plate to neutralize the shear forces across the fracture site. If necessary, we placed another microplate at the posterolateral side of the distal humerus when the posterior condyle was comminuted. Thereafter, we examined the movement of the elbow joint to ensure that there was no abnormal movement and mechanical obstruction caused by the protrusion of the implant. The accuracy of reduction and hardware placement was confirmed radiographically. The wound was irrigated, and the joint capsule was closed over a drain. The common extensor tendon was repaired to the soft tissue cuff on the lateral supracondylar ridge, and the layers of subcutaneous tissue and skin were closed. After surgery, the patient was placed into an elbow-adjustable brace in a 90° flexion position. Rehabilitation was based on the injury type and recovery, with range motion exercises starting no later than 2 weeks postoperatively (Fig. 1).

### Statistical analysis

To evaluate the data, the median and interquartile range were used. The sign test was used to determine if the occurrence of coronal shear fractures was significantly more common in females than males, and if nondominant extremities were more often involved than dominant extremities. Scores were compared by the Dubberley fracture classification system and epidemiologic data using the Mann–Whitney *U* test. The level of significance was set at *P* < 0.05.

## Results

There were more females (35/52 or 67.3%) than males (17/52 or 32.7%) in this study. The average age was 40.4 years (range, 23–62). All fractures were closed. Each fracture was an isolated injury with no associated neurovascular injury. All injuries were the result of trauma (due to a fall), and the non-dominant side was affected 69.2% (36/52) of the time. All patients sustained the fracture after landing on their outstretched upper extremity. The most common type of injury was the fall from the ground (31/52 or 59.6%), followed by the fall from the stairs (9/52 or 17.3%), from the bike (8/52 or 15.4%), and from the roof (4/52 or 7.7%). Based on the AO/OTA classification system [12], 16 (30.8%) cases were type B3.1 and 36 (69.2%) cases were type B3.3. According to the Dubberley classification system [13], there were 11 (21.2%) patients with IA fractures, 5 (9.6%) patients with IB fractures, 16 (30.8%) patients with IIA fractures, 4 (7.7%) patients with IIB fractures, 13 (25.0%) patients with IIIA fractures, and 3 (5.8%) patients with IIIB fractures. The mean time from injury to surgery was 5.8 days (range, 2–9 days).

All patients received follow-up, lasting for 12–24 months (average, 17.6 months). The radiographic review of images revealed an anatomic reduction in 44 (84.6%) cases and a near-anatomic (less than 2 mm displaced) reduction in eight (15.4%) cases. All fractures united by 1-year follow-up with an average of 13.6 weeks (range, 12–16 weeks). There were three cases (5.8%) of Broberg and Morrey grade 1 degenerative arthritis, two cases (3.8%) of Brooker grade 1 heterotopic ossification, and three cases (5.8%) of hardware loosening or breakage without secondary fracture displacement. Seventeen (32.7%) patients opted to have the internal implants removed after healing of the fracture, and five out of 17 patients experienced discomfort of the elbow joint.

**Fig. 2:**
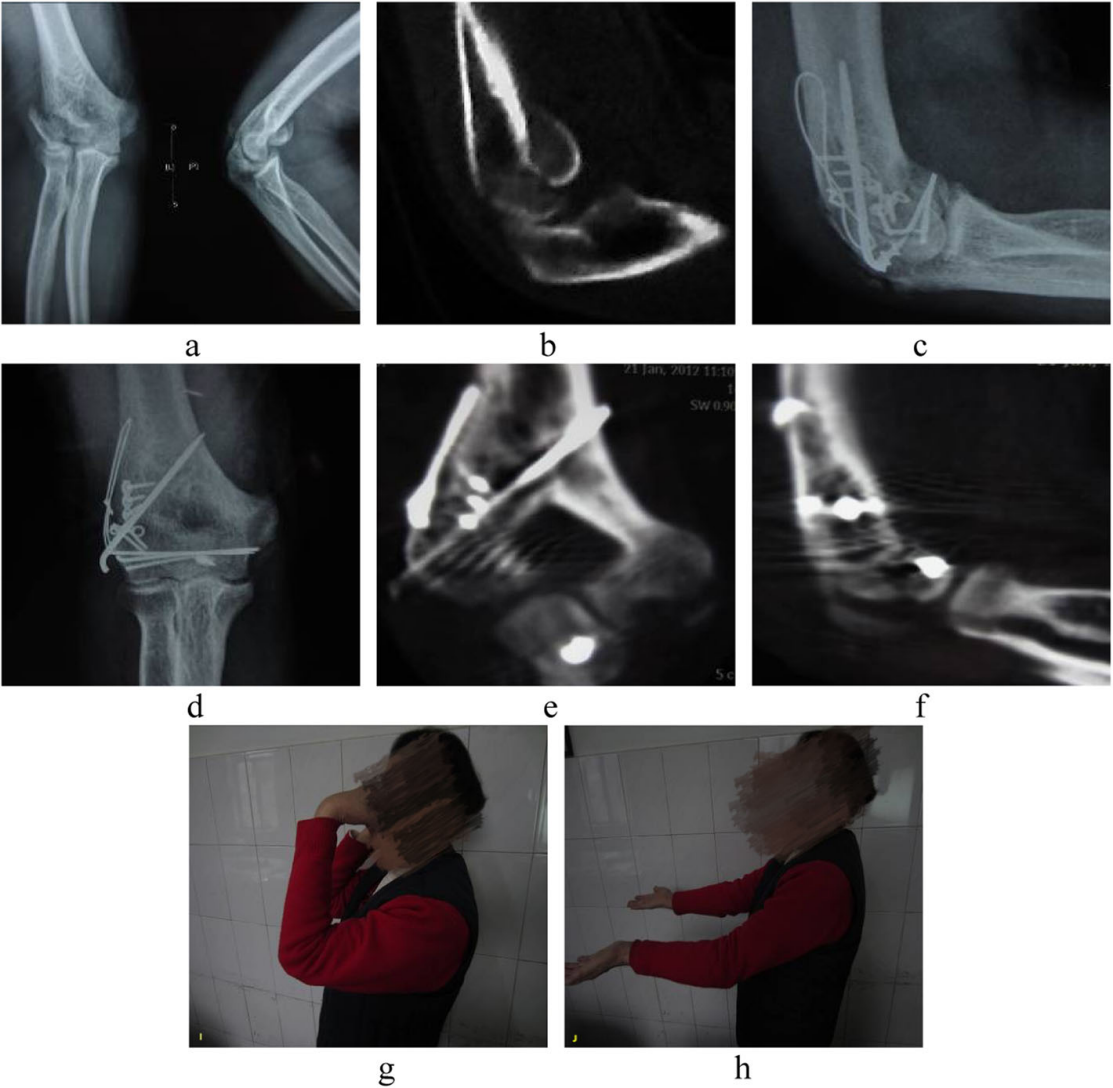
Female, 62 years old, preoperative anteroposterior and lateral radiographs of capitellum and trochlea fractures caused by falling injury from stairs (**a**); preoperative CT showing the capitellum and trochlea fractures and intact posterior condyle (**b**); X-ray radiographs (**c**, **d**) and CT scans (**e**, **f**) 2 days after operation showing the reduction and fixation of the capitellum and trochlea , as well as the good restoration of articular surface of the distal end; functional appearance 1 month after operation showing satisfactory elbow function (**g**, **h**)

The average range of movement of the elbow joint at the last follow-up was 3° (0–15°) for extension and 136° (90–150°) for flexion, with a mean loss of flexion and extension of 9° and 3°, respectively, compared with the contralateral side. No substantial loss of forearm rotation was noted in any of the patients. The average MEPS score was 90.6 (range, 60–100), with 36 excellent results, 11 good results, and five fair results (excellent/good rate: 90.4%). However, the average MEPI score of seven (13.5%) patients with posterior condylar comminution was 71.4, which was significantly lower than the other 45 (86.5%) patients (average MEPI score was 93.6, *P* < 0.05) (Fig. 2).

## Discussion

The clinical treatment of coronal shear fractures of the distal humerus is an active area of research for several reasons. Firstly, it is difficult to compare clinical efficacies between different treatment methods because of the low morbidity rate. Secondly, morbidity presents a bimodal distribution. Fractures in young patients are often caused by high-energy injuries, which are often combined with associated injuries of the elbow. By contrast, fractures in elderly patients are often caused by low-energy injuries, which can lead to postoperative failures of reduction and fixation. Thirdly, it is difficult to preserve and fix all small pieces of the articular surface when the fractures are comminuted. If all small pieces of the articular surface are not retained, the morphology of the articular surface of the humeroradial joint will be altered, thereby resulting in pain, instability, loss of motion, and traumatic arthritis in some patients [7, 8, 18–20].

Treatment options have evolved from fragment excision and closed reduction/immobilization to ORIF in order to achieve stable anatomic reduction, restore articular congruity, and initiate early motion [21–23]. However, various debates have ensued on which surgical fixation technique is most appropriate, which is crucial to the final outcome [24–26]. Presently, Kirschner wires, cancellous screws, Herbert screws, and Acutrak Mini screws are commonly utilized for rigid fixation using the fracture compression technique [27, 28]. Ashwood et al. contended that the maintenance of rigid fixation after fracture reduction, and the stability of the elbow joint are very important [29]. They further stated that the small fragments should be used for reduction and internal fixation. However, Sano et al. considered that if the screws were placed in the small fragments, it would be difficult for the screw threads to traverse the fracture line, resulting in failed fracture compression [30]. Therefore, this may lead to functional disabilities of the elbow joint, because patients cannot rehabilitate elbow joints at an early stage without rigid fixation, especially for patients without access to standard rehabilitation.

Therefore, the anti-sliding plate technique, which is based on the use of Kirschner wires and screws, was utilized for all 52 patients in this study. Our technique has several advantages over previous methods of fixation. Firstly, it can maintain the integrity and continuity of the comminuted fracture of the articular surface by fixation with Kirschner wires and allow for interfragmentary compression with lag screws. Secondly, fixation with a microplate has an effect similar to that of the anti-sliding technique, and it can ensure coverage of the comminuted fragments to provide more stability for small fragments. Previous studies have reported that the greatest force transmission across the radiohumeral articulation occurs between 0 and 30° of elbow flexion [31, 32]. The microplate can also provide an anti-sliding effect for the entire lateral condyle to neutralize the shearing forces that would otherwise contribute to the loss of fixation and to maintain rigid fixation of coronal shear fractures, thereby allowing for the initiation of early range-of-motion exercises.

Jupiter et al. contended that the function of the elbow joint after a coronal shear fracture of the distal humerus is related to the recuperative degree of the normal anatomic structures [33]. All 52 patients were treated with open reduction, with anatomic reduction in 44 cases and near-anatomic reduction in eight cases. We preserved the small fragments as much as possible and strived for the anatomical reduction of these fragments. Lag screws were used to fix the fracture fragments, with temporary fixation with K-wires. Lastly, we maintained the integrity of the articular surface and placed a microplate beneath the articular surface of the humeral capitellum for anti-sliding fixation. Thereafter, intraoperative fluoroscopy with a C-arm was performed to ensure anatomical reduction and rigid fixation of the fracture. Thus, patients were allowed to carry out active and passive activities involving the elbow joint at an early stage.

In summary, both X-rays and CT scans are needed to arrive at a correct diagnosis and classification. The Dubberley classification system, one of the most commonly used systems, focuses on the seriousness of the posterior condyle and the prognosis. The anti-sliding plate was used to obtain rigid fixation on the basis of K-wires and screws after the anatomical reduction of the fracture by the extensile lateral approach. The patients must exercise at the early stage to achieve satisfactory unction of the elbow joint.

The limitations of our study include its retrospective design and the relatively short-term follow-up. This surgical method can be applied to most clinical cases, although the findings should be confirmed in further clinical and biomechanical studies with larger sample sizes.

## Data Availability

We state that data will not be shared because all raw data were used to prepare the figures included in the article.
